# Genomic medicine in the military

**DOI:** 10.1038/npjgenmed.2015.8

**Published:** 2016-01-13

**Authors:** Mauricio De Castro, Leslie G Biesecker, Clesson Turner, Ruth Brenner, Catherine Witkop, Maxwell Mehlman, Chris Bradburne, Robert C Green

**Affiliations:** 1 United States Air Force Medical Genetics Center, 81st Medical Group, Keesler AFB, Biloxi, MS, USA; 2 National Human Genome Research Institute, National Institutes of Health, Bethesda, MD, USA; 3 Walter Reed National Medical Center, Division of Genetics, Department of Pediatrics, United States Army, Bethesda, MD, USA; 4 Air Force Medical Support Agency, Defense Health Headquarters, Falls Church, VA, USA; 5 Law-Medicine center, Case Western Reserve University School of Law, Cleveland, OH, USA; 6 Department of Biomedical ethics, Case Western Reserve University School of Medicine, Cleveland, OH, USA; 7 National Security Systems Biology Center, Asymmetric Operations Sector, Johns Hopkins University Applied Physics Laboratory, Laurel, MD, USA; 8 Division of Genetics, Department of Medicine, Brigham and Women’s Hospital, Partners Personalized Medicine, Broad Institute and Harvard Medical School, Boston, MA, USA

## Abstract

The announcement of the Precision Medicine Initiative was an important step towards establishing the use of genomic information as part of the wider practice of medicine. The US military has been exploring the role that genomic information will have in health care for service members (SMs) and its integration into the continuum of military medicine. An important part of the process is establishing robust protections to protect SMs from genetic discrimination in the era of exome/genome sequencing.

## INTRODUCTION

On 30 January 2015, President Obama announced the Precision Medicine Initiative, a multimillion dollar investment that aims to improve how we treat and prevent disease by focusing upon genetic background, environment and lifestyle.^1^ In the near future, the focus of the Precision Medicine Initiative will be on identifying more effective drug candidates for the treatment of cancer based on the molecular signature of tumours. A second, longer-term component consists of recruiting a national cohort of at least one million Americans, with the broad goal of better understanding disease mechanisms and tailoring therapies for many diseases. Implied in this initiative is that genomics may be used for predictive testing in order to better tailor treatments and offer more targeted preventive services to deliver on the promise of personalised health care.

In the practice of civilian and military medicine, technologies such as single gene and panel testing, chromosomal microarray, exome sequencing (ES) and genome sequencing (GS) are increasingly becoming part of the mainstream tools for diagnosis.^2^ As interest in the use of these technologies expands into screening, the Armed Services are actively engaged in determining when, and how, to integrate the use of large-scale genomic information in the delivery of care for service members (SMs). If ES/GS is eventually utilised for SMs in some capacity as a screening tool, its use will bring special concerns because of the heightened emphasis within the military upon fitness for specific duties, and because of specific issues within the military concerning discrimination.

Recently, members of the genetics community in the military and representatives of civilian academic institutions along with representatives of professional genetic societies met to discuss the opportunities and challenges that the integration of genomic medicine would bring to SMs and the entire beneficiary population in the military. In this commentary, we set out to explore some of these difficult questions.

## USE OF GENETICS AS A SCREENING TOOL IN THE MILITARY: PAST AND PRESENT

Historically, the US military has collected and used genetic information in a number of ways. For example, upon processing through enlisted basic training or officer training school, all SMs must provide a DNA sample to be used for the identification of remains.^3^ These samples can be used in specific circumstances by state, federal and military law enforcement to match DNA found at a crime scene. Other uses of genetic information in the military include screening members for glucose 6-phosphate dehydrogenase deficiency (as it can interfere with the metabolism of certain drugs, i.e., antimalarials) and haemoglobinopathies.^4^ The use of these laboratory test results by the Department of Defense and individual Services has evolved over the years and there are currently Department of Defense- and Service-specific policies that provide guidance around the management of SM who test positive for one of these conditions.

Each branch of Service has its own medical standards for appointment, enlistment and induction, which are different from the standards used to retain a member in the Service. There are more stringent medical standards for certain career fields. Candidates are disqualified from enlisting in the military if they have certain medical conditions. There is a process by which candidates can request waivers to enter the Service despite having a known disqualifying medical condition. In general, having a genetic predisposition for a disease is not considered disqualifying, but displaying the phenotype may be. This process varies by Service and job specialty.

## USE OF GENETICS AS A SCREENING TOOL IN THE MILITARY: THE CASE OF SICKLE CELL TRAIT

A historical example can be found in sickle cell trait (SCT) screening and the story of its implementation in the military. The first policy addressing the issue of increased mortality in carriers for SCT came to light in 1969. The US Navy documented the death of four recruits who perished while undergoing training at moderate altitude (>4,060 feet). These individuals were known to be carriers for SCT. Following this event, the Navy instituted screening for SCT for all recruits, and in 1970, the Military Aviation Safety Subcommittee enacted several restrictions on SM known to be trait carriers, including restricting their activities in aviation, diving, special forces and high-altitude parachuting.^5^ This decision was based on the idea that SCT carriers were at increased risk of sudden cardiac death in the context of rigorous physical training.^6^ In 1981, in the face of increasingly conflicting evidence for a link between SCT and increased mortality, the Department of Defense required the Services to drop the restrictions but to continue screening for the trait, reflecting the ambivalence of the available evidence. In the mid-1990s, following the deaths of three Air Force recruits known to be carriers, the Armed Forces Epidemiology Board revisited the issue. After finding limited evidence supporting a link between the trait and mortality, they recommended against routine screening for SCT and instead recommended improved implementation of heat injury prevention and continued research into a causal link between cardiac events and SCT. In 1996, the Under Secretary of Defense for Personnel and Readiness release a memo stating, ‘Testing for SCT should not be mandated for military accessions’.^7^

Currently, all Services screen potential recruits for a personal history of haemoglobin disorders during the initial exam at military entrance processing stations. Although having a history of sickle cell disease does disqualify a candidate from enlistment in the armed forces, the Services do not currently consider SCT a disqualifying condition. Rather, recruits with SCT are counselled to avoid exertional collapse by gradually increasing activity, maintaining adequate hydration, ensuring proper rest between workouts, avoiding exercise while ill and avoiding low-oxygen and increased-air-pressure environments.^8^ As an alternative, some recruits can opt out of their active duty service commitment and receive an administrative discharge.

The story of sickle cell screening in the Armed Forces has been a complex and contentious one. The Services have had to grapple with related issues of genetic and racial discrimination (as sickle cell is considerably more prevalent among African Americans). It is interesting to note that many civilian groups have publicly released statements against universal screening programmes for SCT in athletes, including the American Society of Hematology, the Sickle Cell Disease Association of America and the US Department of Health and Human Services.^5^

## FUTURE USE OF GENETICS AS A SCREENING TOOL IN THE MILITARY

Although the military community at large will likely benefit immensely from the President’s long-term Precision Medicine Initiative as it relates to the information derived from large Americans cohorts, there may be specific applications of genomic screening in the Armed Forces that cannot be readily extrapolated from civilian studies. Potential benefits of genomic testing in the military could include ensuring troop readiness, optimising performance and in some cases reducing morbidity and mortality. But with these benefits come many challenges. It may be difficult to strike a balance between identifying a deleterious genetic variant to reduce risk and enhance mission effectiveness without adversely impacting the member’s career.

Commanders traditionally have greater access to medical information of SMs under their command than is allowed among civilian employment supervisors. It is important to note that the access to medical information is kept to the minimum required to inform determinations of fitness for duty and safety, and that this is done in conjunction with medical personnel based on established standards. Policies for such access to the genetic information of SMs will need to be developed in ways that can be demonstrated to effectively enhance mission readiness. A clear, rational and reasonable threshold must be developed for determining whether, and how, genetic information is to be used in a screening capacity.

Although there are currently no plans in the military for instituting routine or mandatory genomic screening in addition to glucose 6-phosphate dehydrogenase deficiency and SCT, in specific circumstances there may be additional incentives beyond those in the civilian world to expand the use of genetic information in military members. For example, for highly demanding, high-risk positions such as Special Forces or Special Operations, exposing an at-risk SM to a precipitating situation (i.e., exertion, heat, austere environment or toxic substances) could lead to significant morbidity, loss of manpower, decrease in mission effectiveness and ultimately increase in the cost of human capital and resources for the Armed Forces. For example, individuals carrying mutations in the *RYR1* gene are more susceptible to heat-related injuries and syncope.^9^ In extremely hot environments, *a priori* knowledge of the patient’s genotype could reduce the likelihood of developing exertional rhabdomyolysis by limiting exposure to strenuous physical activity and heat. There may be benefits to the use of genetic information in screening selected members for highly penetrant cardiovascular disease variants that could predispose to increased morbidity and mortality with exposure to heavy exertion. Much work has been done in elite athletes, who endure gruelling training sessions to reach their peak potential. For example, variants in desmosomal genes associated with arrhythmogenic right ventricular dysplasia^10^ or in one of the 15 genes known to cause long QT syndrome, have been reported as a cause of sudden cardiac death in athletes.^11^ In addition, other groups have found an increased risk of stress fractures in carriers of variants in genes in the *RANK/RANKL/OPG* pathway.^12^ These associations and the questions that they raise are becoming more relevant as genomic testing moves from the realm of diagnosis and treatment of disease and into the realm of predictive testing, with its use in healthy cohorts for identification of potentially actionable conditions, pharmacogenomics variants and carrier status in individuals of reproductive age.

An important issue that will need consideration is how best to approach the selection of variants to screen for. One solution would be to select for variants thought to be penetrant and clinically actionable, akin to the 'American College of Medical Genetics and Genomics’ working group on incidental findings in which variants were selected partly based on a consensus on the variant’s pathogenicity, penetrance and clinical implications.^13^ For the adaptation to military medicine, special emphasis could be given to variants known to affect an individual’s ability to carry out physically demanding tasks (adult-onset myopathies for instance) or situationally relevant variants (e.g., variants associated with high-altitude pulmonary oedema). Civilian studies may provide a general framework to identify potential variants; sequencing of a cohort of individuals enriched for a specific phenotype (exercise-induced myalgia for instance) could be used to identify variants of interest. Studies performed in military settings will need to be carried out to test the feasibility and acceptability of such frameworks in identifying relevant variants.

## THE MILITARY AND GENETIC DISCRIMINATION

The Genetic Information Non-Discrimination Act does not apply to the military, raising concerns about the potential for genetic discrimination,^14^ but there are policies in place that govern how genetic information is managed in the military health-care system. Changes made to the law in 2008 and 2009 altered a previous policy that had denied health and disability benefits to SMs who experience injury or illness during their time of service if the condition was ‘congenital or hereditary.’ Now, SMs are entitled to compensation and benefits so long as there is no clear and unmistakable evidence that they had a hereditary or congenital disease at the time of enlistment, or if the disease was aggravated by their service.

A variant found in genetic testing cannot result in any action unless that individual suffers symptoms during their time of service and those symptoms limit the member’s ability to carry out their duties. For example, if a service member undergoes diagnostic ES/GS and an incidental variant in a familial cancer syndrome is found, this individual will receive all appropriate medical care but no medical board will be convened unless the syndrome-associated cancer develops. No commander can override this policy. Actions taken after symptomatic manifestations of a genetic condition are highly individualised and depend on a number of factors; the condition in question, length of service, duty assignment and, length of service and other mitigating circumstances. For example, a pathogenic variant in an inherited cancer syndrome would imply enhanced screening that would not necessarily impact an SM’s duties; the same cannot be said of a pathogenic variant found in a long QT syndrome-associated gene that could impact duty performance and for which restrictions could be placed (if indicated based on clinical judgment and/or future policy). This is a significant issue that needs to be addressed in the pre-test counselling of any SM.

An additional layer for service members are the Equal Opportunity directives that include genetic information as a protected category, to wit, Air Force instructions states that ‘it is against Air Force policy for any Airman, military or civilian, to unlawfully discriminate against, harass, intimidate or threaten another Airman on the basis of race, colour, religion, sex, national origin, age, disability, reprisal or genetic information’, thus in the Air Force it is unlawful to discriminate against an SM based solely on a the presence of a genetic variant.

Although employment discrimination based on genetic information has been rare in the civilian world,^15^ there are unique aspects to the military that make it more likely to occur. In the near future there could be use for genomics and other technologies to help stratify risk and vulnerability related to specific occupations. In the military, in contrast to the civilian world, the welfare of the individual is subservient to the unit, mission and country.^16^ Because commanders (the hierarchy above the SM) have duties that may reduce an SM’s autonomy, policies need to be in place to ensure that the risks of genomic testing, including loss of privacy and potential breaches of confidentiality, are outweighed by military necessity. This is especially important in the case of genomic testing for non-disease characteristics. The military has a legitimate interest in obtaining information about warfighters’ physical and mental abilities, including genomic information, but only if the genomic test is a valid indicator of what it purports to show and the information is necessary in order to carry out the mission.^15^

An interesting question that may arise in the future is what to do with an SM’s ES/GS data. Precedent exists in the military to the use of genetic information outside of its original intended purpose (i.e., DNA repository). Consent forms used in the collection of genetic information from military members (clinical and research) should be clear on the intended use for the data and give SM the opportunity to opt out of future research. It is also important to note that an SM can request any existing DNA samples in the Department of Defense repository be destroyed after completion of military service.

## INCIDENTAL FINDINGS

Genetic tests such as chromosomal microarray, ES or GS that can yield variants other than the intended target (incidental or secondary findings) have not yet been addressed by existing military regulations. Although robust regulations are in place to protect SMs from being discharged or medically boarded from the Service based solely on genetic information obtained through diagnostic testing, the unintended consequences of testing performed in healthy individuals undergoing genomic screening for disease susceptibility, pharmacogenomics or carrier screening have not been fully elucidated. Secondary findings associated with ES/GS include several types of genomic findings, ranging from rare and more highly penetrant risk variants to more common variants that are associated with very modest risks. On the basis of the studies to date,^17,18^ 1–3% of SMs would likely be found to have a secondary finding in a gene associated with a dominant, actionable medical condition, mostly genes associated with rare forms of hereditary cancer and cardiac disease. Almost everyone will carry variants for rare recessive conditions,^19^ and everyone will have some combination of common variation that may be associated with common diseases (such as diabetes) or with pharmacological effectiveness or safety. For all unaffected individuals, these would be considered susceptibility variants, but would not be diagnostic in any way. Individuals found to have variants associated with an actionable dominantly inherited condition would be referred to the appropriate specialist to review the personal and family history, looking closely for potential manifestations of the disorder.^2^ Only if symptoms were to develop for a condition that was duty limiting would evaluation by a medical board be necessary. Thus, it would be the condition and its manifest symptoms that would be evaluated as potentially service limiting, rather than the risk variant. Thus, if these policies were fully followed, secondary findings could be discovered when there was sequencing for indication, and even genomic susceptibility testing could be performed, without necessarily affecting the disposition of the SM.

It is important to note that for diagnostic testing, SMs are afforded the same options as civilians, i.e., data on pathogenic incidental findings can be rejected by the patient, allowing for the option to opt out of knowledge of any incidental findings.

## CHALLENGES TO IMPLEMENTATION

Apart from the scientific and medical possibilities and policies regarding genomic testing there are the challenges of practical implementation of genomic screening. In the civilian population, physicians report low confidence in their ability to use genomic data in the care of patients^20^ and this is likely true in the military as well. In the military, as in the civilian sector, there are not enough geneticists or genetic counsellors to fill the need for conventional pre- and post-test genetic counselling in a world where genomics is fully integrated into health care. Owing to this, and to the wide geographic distribution of military bases, other approaches are being explored in the delivery of counselling and appropriate reporting of genetic results. This will include efforts to educate both primary care and specialty providers at all levels and the presence of remote assistance from a geneticist and/or genetic counsellors via telemedicine. In the end, genomic medicine is not exceptionally different from other branches of medicine;^21^ its integration into the larger continuum of military medicine will depend on appropriate provider training and continued support from specialists and the health system at large.

Military medicine faces similar opportunities and challenges in adopting genomic medicine as the civilian world, but with some additional challenges. Because of the extreme physical challenges present in some military roles, there may be specific circumstances in which genomic screening information could be utilised to enhance force readiness, but these circumstances should be distinguished from the concept of screening the force at large, where incomplete penetrance and variable expressivity for most variants, along with the absence of data on health outcomes, do not support such implementation. Although the Genetic Information Non-Discrimination Act does not cover the military, there are protections in place to guard SMs against discrimination for asymptomatic risk variants found in genomic screening tests. It is also important to point out that once an SM leaves the military, the Genetic Information Non-Discrimination Act protects them from discrimination that might accrue from genomic risk factors discovered in the military.

Future progress on integrating genomic medicine into the military will depend on forging sound policies, which in turn will rely on obtaining strong evidence from empirical studies. A significant difficulty is the lack of data on the degree of risk conferred by genomic variants (i.e., penetrance and expressivity), particularly in the absence of family history; the degree of clinical utility conferred by the discovery of such variants; and the cost–benefits of any form of screening.^21^ In addition, we lack systematic evidence about how such information might be used by SM. Only actual, real-world data, collected in military settings from SM based on its use by military health-care providers and commanders, can help address these challenges more concretely. Thus, there is an urgent need for more empirical genomic studies in the military, along the lines of what our civilian partners are carrying out with large-scale federally funded research collaborations (http://genome.gov/27551170). With such evidence in hand, we can begin answering the question of how to best integrate genomic medicine into the clinical practice of military medicine and its effect on the operational environment not just in the United States but in other countries as well.
